# Impaired memory for cooperative interaction partners in borderline personality disorder

**DOI:** 10.1186/s40479-020-00137-3

**Published:** 2020-10-01

**Authors:** Inga Niedtfeld, Meike Kroneisen

**Affiliations:** 1 Department of Psychosomatic Medicine and Psychotherapy, Central Institute of Mental Health, Medical Faculty Mannheim / Heidelberg University, J 5, 68159 Mannheim, Germany; 2 Department of Psychology, University of Koblenz-Landau, Fortstraße 7, D-76829 Landau, Germany

**Keywords:** Borderline personality disorder, Social cognition, Source memory, Cooperation, Trust

## Abstract

**Background:**

Interpersonal disturbances in Borderline Personality Disorder (BPD) have been attributed to a negativity bias in social cognition. Adding to this literature, we experimentally tested whether those with BPD show altered memory for cooperative versus non-cooperative interaction partners.

**Methods:**

In a source memory paradigm, 51 female BPD patients and 50 healthy controls (HC) played a trust game with 40 different female target characters (trustworthy vs untrustworthy). In a subsequent surprise memory test, participants had to recognize those target individuals (vs distractor pictures), and had to recall whether they had shown cooperative behavior during the trust game. We hypothesized that BPD patients have better memory for uncooperative interaction partners as compared to cooperative interaction partners, and that a-priori expectations of untrustworthiness would influence recall.

**Results:**

During the trust game, BPD individuals invested lower amounts of money than HC for trustworthy targets, but no differences were found for untrustworthy targets. During the memory test, BPD patients had significant difficulties to remember cooperative targets, as compared to HC. More specifically, those with BPD indicated more often than HC that they had not previously interacted with cooperative targets of the previous trust game. We did not detect any differences between BPD and HC in source memory, or with regard to the effects of trustworthiness expectations.

**Conclusions:**

The observed tendency to forget cooperative interaction partners in BPD is possibly caused by dysfunctional cognitive schemas. At the same time, it might also corroborate patients’ assumptions that others are untrustworthy, thereby fuelling interpersonal disturbances in BPD.

## Introduction

Interpersonal Problems are considered one of the most stable symptoms in BPD [1, 2]. Relatedly, dysfunctional behavior or suicide attempts accumulate in the course of problematic interactions [3, 4]. Improvements in social functioning are comparatively weak in those with BPD [5], and even remitted BPD patients are characterized by low social functioning [6]. Recent research on social interaction in BPD suggests that impairments in interpersonal relationships can be related to reduced trust [7], deficits in cooperation [8], and alterations in social cognition [9].

Importantly, biases in social cognition have been reported in many mental disorders other than BPD, including depression [10], eating disorders [11], and social anxiety disorder [12]. In our study, we chose to investigate a sample of BPD patients to study the role of memory processes in patients with interpersonal problems, but without the expectation that these effects are specific for BPD. Nevertheless, we chose BPD as an exemplary population, since patients show a high level of interpersonal dysfunction across several domains [13], and there is a substantial body of previous studies on social cognition in BPD [9], enabling us to deduce hypotheses. By sharing our experiment and data, we hope that our experimental paradigm might be easily adopted by future research on memory biases in other patient populations, and across disorder categories [14].

In addition to alterations in social cognition [9], selective memory for negative social information might be an important factor fuelling interpersonal problems in BPD. Reviewing studies on long-term memory in BPD, Baer et al. [15] summarize findings of selective memory for negative words. For example, those with BPD showed marked problems to forget stimuli that were related to rejection and abandonment (e.g. lonely, misunderstood, cruel, uncaring) [16] in a directed forgetting task. Studies on autobiographic memory in BPD also revealed that patients have increased access to specific negative memories [17]. Furthermore, patients more frequently reported situations of abandonment or deliberate harm by others [18].

In search for factors causing selective memory for negative information, studies on cognitive schemas in BPD show that patients tend to view the world as dangerous and malevolent, and themselves as powerless and vulnerable [19, 20], expecting that they will betrayed and abandoned by others [21]. Therefore, one may assume that a tendency to expect that other persons are untrustworthy in general [22–24] might lead to an increased processing of negative social information in memory. In a range other mental disorders, increased memory for disorder-related stimuli was found [25, 26], and attributed to schema-congruent processing. If schema-congruent processing applies to memory function in BPD, a better memory for untrustworthy individuals can be expected. Finally, whenever unsure, those with BPD should have a tendency to guess that others behaved untrustworthy, because this aligns with dysfunctional cognitive schemas.

However, no study has experimentally tested whether patients with BPD show altered memory for cooperative versus non-cooperative interaction partners. This is important, because successful social interactions are based on the ability to differentiate cooperative from uncooperative interaction partners (for an overview, see Fehr and Gächter [27]). Since reciprocity is often a delayed process, memory research has emphasized the importance for the individual to correctly remember information about the trustworthiness of other people [28]. More specifically, individuals have to recognize individuals with which they had previous encounters (i.e. item recognition, “I have seen this person before”), and have to recall previous cooperative or non-cooperative behavior (i.e. source memory, “this person behaved trustworthy”) at the same time. Studies on source memory in healthy populations showed that untrustworthy behavior was remembered better than trustworthy actions [28, 29], but this effect was mainly due to a violation of initial expectations [30–32].

With regard to BPD, drawing on previous findings of deficits in trust and cooperation [8], as well as a general tendency to selectively remember negative information [15], we expected that those with BPD show better memory for uncooperative interaction partners as compared to cooperative interaction partners, and that this effect is larger in magnitude than the negativity bias that is seen in the general population [33]. Additionally, we assumed that dysfunctional cognitive schemas in BPD [20] might also have an impact on a-priori trustworthiness expectations and therefore might influence memory processing. If patients anxiously expect other people to let them down, untrustworthy interaction partners should have a high relevance for those with BPD, especially when the interaction partners initially made a positive first impression.

By means of the source monitoring paradigm, it is possible to test memory performance for uncooperative and cooperative interaction partners, as well as the effect of a-priori trustworthiness expectations (e.g. [30]). Therein, positive and negative expectations are manipulated a-priori in order to test memory performance for congruent as well as incongruent information. More specifically, participants are presented with photographs of target persons that evoke a positive or negative first impression, and then aquire information about the cooperativeness of the respective target (either by own experience in an economic game or via short vignettes). Recent work on source memory for faces of cooperators and cheaters has shown that source memory was modulated by participants’ positive or negative expectancies [30]: Depending on the environment, healthy subjects had better source memory for cheaters or co-operators. Therefore, the authors concluded that source memory of healthy individuals is adaptive in the sense of maximizing diagnosticity. In a cooperative environment, it is sufficient for them to remember the few cheaters (and avoid them) and vice versa for the non-cooperative environment.

In the current study, we used the source memory paradigm as established by Bell, Buchner [30] to experimentally test item memory and source memory for cooperative and uncooperative interaction partners in BPD, and to investigate the influence of a-priori expectations. Importantly, while all previous studies on item memory in BPD employed word lists that were intentionally learned by the participants, we apply a modified single-round trust game with multiple interaction partners to experimentally investigate incidental learning.

We hypothesized that, in concordance with dysfunctional cognitive schemas, (1) patients with BPD would show better memory for uncooperative interaction partners than for cooperative interaction partners, and that this negativity bias is more pronounced than in HC. With regard to a-priori expectations, (2) interaction partners that seem trustworthy but show uncooperative behaviour should be especially relevant for those with BPD, and therefore should be remembered more precisely in BPD than in HC. Finally, on the basis of schema-congruent processing, we (3) expected those with BPD to show a more pronounced guessing bias towards non-cooperativity in unknown interaction partners as compared to HC.

## Methods

### Selection of stimulus material

Since it was unclear whether the manipulation of expectancy would be successful in BPD patients or might be distorted due to evaluation biases [20], we decided not to rely on photographs that were used in previous studies with the source memory paradigm in healthy subjects [30]. Thus, we conducted a pilot study and recruited a web-based sample of 156 subjects from social networks as well as BPD-specific websites. They provided demographics, filled out the Borderline scale of the Verhaltens-Erlebens-Inventar (VEI, [34]), which is the German adaptation of the Personality Assessment Inventory (PAI, [35]). Afterwards, they were asked to rate a subsample of 60 pictures from a set of 280 pictures of caucasian female subjects on trustworthiness (on a likert scale ranging from 1 = not trustworthy to 8 = very trustworthy). Based on these ratings, we selected a set of 80 pictures with high and low trustworthiness, while ensuring that each had similar trustworthiness ratings when comparing those with low BPD features to those with high BPD features. Of these 80 pictures, we built two subsets of 40 faces (20 trustworthy and 20 untrustworthy faces each, matched for mean trustworthiness). The chosen picture sets were randomly assigned during the experiment to serve as target material, or distractor material for the memory test only. A detailed description of the pilot study and data are available in the Open Science Framework (OSF) repository, https://osf.io/hvf42/).

### Sample characteristics

For the source memory experiment, we invited 52 healthy female participants and 52 females with Borderline Personality Disorder according to DSM-IV [36]. Healthy subjects were recruited by newspaper advertisement, and patients with BPD were recruited by the research unit of the Department of Psychosomatic Medicine, Central Institute of Mental Health (CIMH) in Mannheim. The study was approved by the ethics committee of the Medical Faculty Mannheim, Ruprecht-Karls-University Heidelberg (protocol no. 2013-654 N-MA).

To assess psychopathology, trained psychologists performed structured clinical interviews, the German versions of the International Personality Disorder Examination [37] and the Structural Clinical Interview for DSM-IV Axis-I [38]. Borderline patients had on average 1.25 current comorbid Axis I diagnoses. Exclusion criteria for patients were current schizophrenia, bipolar disorder, substance abuse, a current severe depressive episode, as well as current psychotropic medication within 6 weeks prior to the experiment. Since the current study was part of a larger project on social information processing in BPD (http://gepris.dfg.de/gepris/projekt/256645687?language=en), several self-report questionnaires were used. Since we had no hypotheses with regard to the relation of self-reports and the effects in the reported task, and for the sake of completeness and transparency, we report all self-report data for the current sample in the OSF repository (https://osf.io/hvf42/).

The final sample for statistical analyses consisted of 51 patients with BPD and 50 healthy controls, which did not differ significantly in age (BPD = 28.67(6.79); HC = 29.5(9.14); t_(99)_ = 0.521, *p* = .604) or level of education (Mann-Whitney U-Test; *p* = .283). Data of three participants were excluded from all analyses: One healthy control had a positive drug urine test, one patient had taken psychotropic medication at the day of the study, and one healthy control fulfilled criteria for an anxiety disorder.

### Source memory task

To test memory for cooperative and uncooperative interaction partners, we adopted a validated source memory paradigm [28, 30]. During the first phase of the laboratory experiment, 20 trustworthy and 20 untrustworthy facial photographs were presented in the context of a single-round trust game with multiple players [28, 30]. During the game, subjects were supposed to learn from their own experience whether the respective target person showed either cooperative or non-cooperative behaviour. Thus, the experimental design comprised two independent variables: A-priori expectation (trustworthy vs non-trustworthy targets, as evaluated via web-study) and behaviour (half of the targets showed cooperative behaviour in the trust game, the other half of the target persons cheated) were combined as within subject factors. For each participant, half of the trustworthy and half of the untrustworthy targets were randomized to either the cheater or the cooperator condition. Consequently, the learning phase comprised 40 trials, with 10 trials each for trustworthy cooperators, trustworthy cheaters, untrustworthy cooperators, and untrustworthy cheaters.

First, the participants were informed that they would “play for money with different people” and that they “will receive 1/3 of this money at the end of the study” (in addition to participant fees of 12€ per hour). They were instructed that “during each round, both you and your opponent will have the opportunity to decide how much money you want to use from your account (initially 450 cents). You can decide between an investment of 0 Cent, 15 Cent or 30 Cent. After you have determined your investment, the investment of the other player is also displayed on the screen (i.e. 3.5 seconds later). Then, a bonus is added to the sum of both investments, consisting of 1/3 of the total sum. At the end of each round, the total money is split by two, with each player getting half of the total money back into his account. The best strategy to win money is to invest as much money as possible if you expect the other player to make a big investment, and to invest little or nothing if you expect the other player to make a low investment.” Importantly, in trials with a cooperative target, the other player returned more money than the participant invested (+ 15 cent). However, in rounds where the participant had chosen the highest investment (30ct), a cooperative target invested the same amount. In trials with uncooperative targets, the other player invested nothing (0 cent).

After participants played 40 trials of the trust game, they were informed that they now had to complete the second part of the experiment, a surprise memory test. During the memory test, we presented 80 faces, half of them old (i.e. targets from the trust game; stimulus set 1 or 2, depending on the random assignment in the first phase) and half of them new (distractor items from the other stimulus set, no previous encounters, 20 trustworthy and 20 untrustworthy faces). For each target, participants first had to choose whether they “know this person from the first part of the study” via mouse klick on one of two buttons (differentiating old and new items = *item memory*). Whenever participants endorsed that they recognized the target, they subsequently had to indicate whether this target had behaved “fair” or “unfair” during the trust game (remembering behaviour of the target = *source memory*).

### Statistical analysis

To test our hypotheses, we calculated multilevel models (mixed effects models), predicting each dependent variable with the fixed factors group (BPD vs HC), a priori trustworthiness of the target (trustworthy vs nontrustworthy^1^), behaviour of the target in the trust game (Cooperator vs Cheater), as well as their interaction. Additionally, we entered a random factor (intercept) for each participant. Multilevel analyses were conducted in R [39] using the lmer function from the lme4 package [40], and *p* values were computed using the lmerTest package [41]. In the case of significant (*p* < .05) results, effect size Cohens *d* [42] was computed from mean values and reported. Finally, we set up a hierarchical multinomial processing tree (MPT) model [43], and estimated memory parameters as well as goodness-of fit tests with and estimated memory parameters as well as goodness-of fit tests with MultiTree [44].

## Results

The datasets supporting the conclusions of this article as well as complete data on self-reports are available in the OSF repository (https://osf.io/hvf42/). For basic descriptive statistics, see Table 1.

**Table 1:** Descriptive Statistics. For complete self-report data, see online repository (https://osf.io/hvf42/)

	BPD (*n* = 51)			HC (*n* = 50)			Cohens *d*
	AM	SD	n	AM	SD	n	
Age	28.67	6.79		29.50	9.14		0.10
Education							
*Abitur*			32			36	
*Realschulabschluss*			16			13	
*Hauptschule*			3			1	
BSL-23	1.71	0.11		0.66	0.14		3.93***
PAI-BOR	50.26	13.68		7.26	7.01		5.13***
RSQ	16.86	4.4		6.17	2.62		2.8***
PANAS negative affect	1.58	1.06		0.47	0.12		1.75***
PANAS positive affect	2.22	3.09		0.61	0.68		1.35***

*BSL* Borderline Symptom List BSL-23 [45], PAI-BOR = borderline personality features, personality assessment inventory [46], *RSQ* Rejection sensitivity questionnaire [47], *PANAS* Positive and Negative Affect Schedule [48]; ***p < .001, **p < .01, *p < .05.

### Trust game behaviour

During the first phase of the experiment, participants had to decide how much money they wanted to invest in each round of the trust game (0, 15, or 30 cent). We ran exploratory analyses and predicted investments by the a-priori trustworthiness of the target and the diagnostic group. We found a main effect for trustworthiness, with higher investments for trustworthy targets, and an interaction effect for group and trustworthiness. Post-Hoc pairwise comparisons suggest that whenever targets were trustworthy, BPD invested lower amounts of money (AM = 15.4 cents, SD = 5.35) than HC (AM = 17.39 cents, SD = 4.90) (Z = 2.252, *p* < .05, *d* = 0.39), but groups did not differ significantly regarding their investments for untrustworthy targets (BPD: AM = 8.03 cents, SD = 4.14; HC: AM = 8.57 cents, SD = 5.57).

### Item memory

**Fig. 1:**
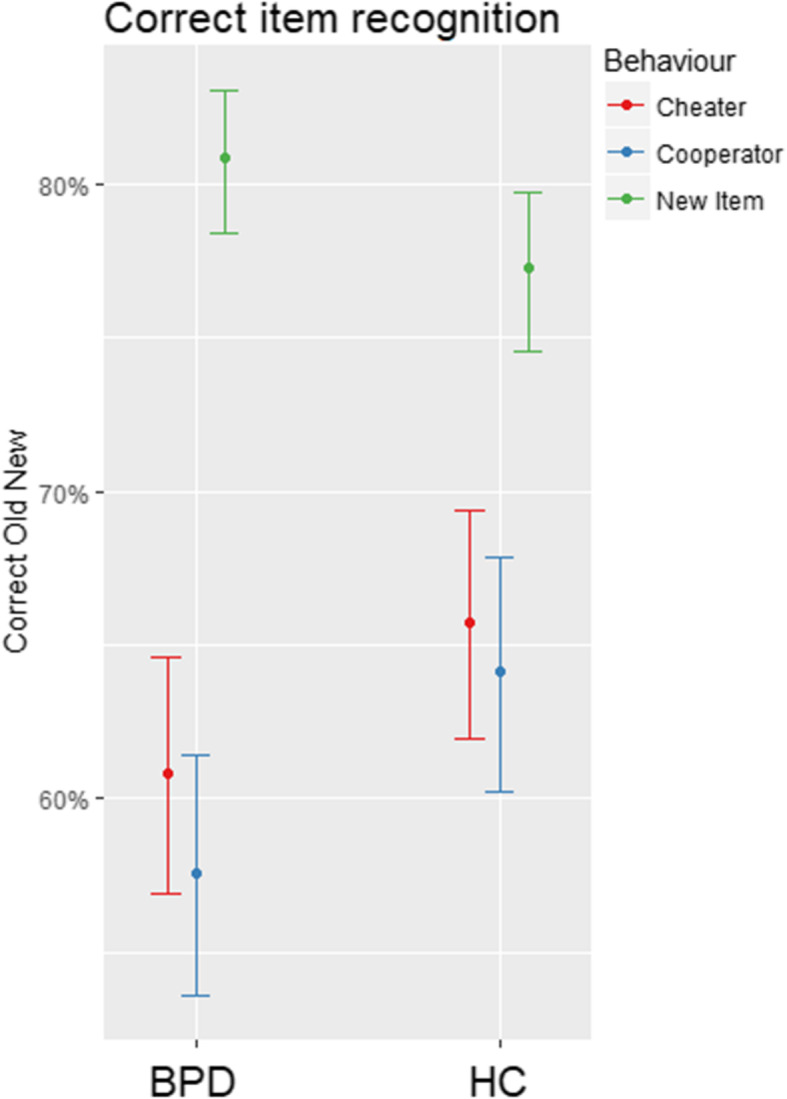
Predicted Probabilities for Correct Item Recognition (estimated mean, standard error), dependent on Group (BPD vs HC), Behavior (Distractor Item, Cheater, Cooperator), and a-priori trustworthiness (trustworthy vs untrustworthy)

Testing our first and second hypotheses, we predicted correct item memory (i.e. correctly classifying old targets as well as new distractors) by the factors group, trustworthiness and behaviour. We observed a significant main effect for behavior, pointing to more correct classifications for distractor items than for old target items, and a significant main effect for group, pointing to somewhat lower item memory in BPD as compared to HC (mean % correct classifications in BPD = 69.46 (9.85), HC = 70.68 (7.39), *d* = .14). Finally, there was a significant interaction effect for group and behavior. Partly confirming our first hypothesis, post-hoc pairwise comparisons pointed to lower correct recognition of targets that behaved cooperatively in the trust game in those with BPD versus HC (BPD = 57.25% (21.82); HC = 63.80% (17.54); *d* = .33). For cheaters, we did not observe significant differences between BPD (AM = 60.39% SD = 18.05) and HC (AM = 65.40%, SD = 16.78). Additionally, post-hoc tests pointed to better item memory for BPD as compared to HC with regard to new distractor items (BPD = 80.10% (15.77); HC = 76.75% (15.68); *d* = .21). However, as opposed to our second hypothesis, there was no interaction with the a-priori trustworthiness of the targets, since we did not observe a three-way interaction effect (group, behavior, a-priori trustworthiness). For details, see Table 2 and Fig. 1.

**Table 2:** Results for mixed effects logistic regression, prediction of correct item memory (yes/no) by fixed factors group (BPD vs HC), trustworthiness (trustworthy vs untrustworthy), and behaviour (New Item, Old Cheater, Old Cooperator), interaction terms, and a random intercept for each subject

	Correct Old New		
	*Odds Ratios*	*CI*	*p*
*Predictors*			
(Intercept)	4.71	3.90–5.70	**< 0.001**
GroupHC	0.74	0.57–0.97	**0.026**
Trustworthiness (trustworthy)	0.81	0.65–1.01	0.056
Behaviour (Cheater)	0.35	0.28–0.45	**< 0.001**
Behaviour (Cooperator)	0.29	0.22–0.36	**< 0.001**
GroupHC:Trustworthiness (trustworthy)	1.16	0.86–1.58	0.328
Group (HC):Behaviour (Cheater)	1.72	1.22–2.42	**0.002**
Group(HC):Behaviour (Cooperator)	1.94	1.38–2.72	**< 0.001**
Trustworthiness (trustworthy):Behaviour (Cheater)	1.08	0.77–1.52	0.649
Trustworthiness (trustworthy):Behaviour (Cooperator)	1.26	0.90–1.76	0.177
Group (HC):Trustworthiness (trustworthy):Behaviour(Cheater)	0.81	0.50–1.30	0.377
Group (HC):Trustworthiness (trustworthy):Behaviour(Cooperator)	0.72	0.45–1.16	0.175

With regard to our third hypothesis related to false positives in item memory, we predicted false alarms (i.e. new items classified as old) by group and trustworthiness, resulting in a main effect for trustworthiness, but no effects for group or interaction effects. While trustworthy distractors led to increased false alarms, there were no significant differences between BPD (AM = 19.90%, SD = 15.62) and HC AM = 23.25%, SD = 15.52). Therefore, we could not confirm our third hypothesis, that those with BPD would falsely attribute non-cooperativity to unknown persons.

### Source memory

Additionally, we analyzed source memory (i.e. correctly classifying a cooperator / cheater) in all trials where a correct item recognition occurred (i.e. subjects correctly classified the target as an old item). Predicting correct source attribution by the factors group, trustworthiness and behaviour, we observed main effects for trustworthiness and behaviour, as well as a large interaction effect for trustworthiness and behaviour. Taken together, trustworthy cooperators (AM = 38.22, SD = 20.42) were recognized better than untrustworthy cooperators (AM = 23.27, SD = 17.44, *d* = .56), and untrustworthy cheaters (AM = 41.29, SD = 21.48) were recognized better than trustworthy cheaters (AM = 25.74, SD = 16.93, *d* = .57). However, we found no significant differences between BPD and HC, nor any interaction effects with the group factor, i.e. we could not confirm our second and third hypothesis. Mirroring these results, the multinomial processing tree (MPT) model also detected differences in item memory between BPD and HC, but also did not show any significant differences between groups with regard to the source memory parameter d, or guessing biases (see OSF repository for more details).

## Discussion

In the current study, we experimentally investigated memory for cooperative and uncooperative interaction partners, in order to explore possible underlying factors of interpersonal deficits in BPD [1, 2]. Our goal was to investigate whether patients with BPD tend to remember cheaters better than cooperative interaction partners, and this would be influenced by a-priori expectations. We partly confirmed our first hypothesis, that patients with BPD would show better memory for uncooperative interaction partners than for cooperative interaction partners, and that this negativity bias is more pronounced than in HC. Specifically, we found that cooperative targets were remembered worse in the BPD group than in the HC group, but there was no difference with regard to uncooperative targets. In other words, BPD patients stated in 43% of all cases that they did not know a cooperative target, although they interacted with them in a previous trust game. With our experimental design, we cannot differentiate whether prosocial interactions of trustors were consolidated but not recognized, or were never consolidated. We conclude that dysfunctional cognitive schemas relevant with regard to BPD, that others will betray or abandon them [21] or are generally untrustworthy [22–24] might lead to decreased processing of positive (i.e. schema-incongruent) social information in memory.

Against our Hypothesis 2, that targets with high a-priori trustworthiness that show uncooperative behaviour should be remembered better in BPD than in HC, we did not observe any differential effects of a-priori trustworthiness as evoked by the stimulus-material depending on the diagnostic group. Although we initially evaluated our stimuli in a web-based pilot study, and ensured that they lead to positive and negative impressions also in subjects with high BPD features, we think it is possible that dysfunctional cognitive schemas [20, 21] might have overruled these first impressions, thereby diluting the effect of the stimulus material.

With regard to lower item memory for cooperative interaction partners, we think it is especially important to consider that due to our experimental design, those with BPD had comparable possibilities to make a positive experience. In fact, those with BPD made lower investments (around 15 ct) for targets with high a-priori trustworthiness during the game than HC (more than 15 ct). Importantly, this means that those with BPD even had more trials where the co-player repaid a higher amount of money as compared to HC, because whenever one invested 30 ct, the target also returned 30ct. Nevertheless, patients with BPD had difficulties to remember those cooperative targets. Dysfunctional cognitive schemas in BPD might explain this altered item memory for cooperators, apart from a general tendency to rate “new” in the decision phase. Since BPD patients have a tendency to assume that others are uncooperative and hostile [19, 20], and expect others to reject them [47], the experience of another person showing cooperative behavior might violate the maladaptive schema. According to cognitive models of psychopathology (e.g. [49]), new information that is incongruent with a maladaptive cognitive schema is likely to be misinterpreted or forgotten, thereby immunizing the maladaptive schema. Our finding is also in line with other studies on long-term memory in BPD, where schema-relevant information was remembered better than neutral information [15]. Additionally, some recent studies on various aspects of social cognition in BPD likewise point to altered processing of positive stimuli in BPD [50–54]. Therefore, in addition to enhanced processing of negative stimuli, low processing of positive stimuli in BPD might further aggravate the negativity bias in BPD by a marked asymmetry with regard to negative versus of positive content.

With regard to source memory (Hypothesis 3), we did not detect significant group differences between groups regarding a tendency to guess that a target behaved uncooperatively. Additionally, although we found a large interaction effect of trustworthiness and behavior in source memory, the direction of this effect was not in line with previous studies using the same experimental design in student populations [30–32]. As opposed to these previous findings, we found that expectation-congruent behaviour was remembered better with regard to source memory. However, as compared to the mentioned studies, item recognition in the current study was relatively low, with correct classification of old targets in only 60–70% of cases. Therefore, we cannot make conclusions with regard to source memory in BPD, but will have to rely on further research to answer this question.

With regard to cooperative behavior during the trust game, we found that those with BPD invested less money than HC, especially when interaction partners made a trustworthy first impression. This finding extends earlier studies using the trust game in BPD (for an overview, see 8), although a general tendency to invest less money in this game cannot be attributed on trust beliefs alone, but might be influenced by other motives like social welfare and risk aversion [55]. Noteworthy, we found a differential effect in our study, since those with BPD invested lower amounts of money as compared to HC only when targets had high a-priori trustworthiness. As opposed to the experimental manipulation in our study, where cooperative interaction partners always repaid more than they received, low investments in trustworthy interaction partners in real-life interactions might have negative consequences. For example, avoiding to lend money, share a secret, or team up with a colleague may lead to fewer situations where those with BPD can experience cooperative behavior by others, thereby further fueling maladaptive cognitive schemas.

Synthesizing our findings of lower investments regarding trustworthy individuals, and also lower memory for trustworthy interaction partners, our results align with previous findings on social rejection in BPD, especially that patients felt more rejected than HC when they were included [56], and fail to integrate previous experiences of inclusion into subsequent interactions [54]. This seems to suggest that cooperative behavior of others might cause conflicts with their negative mental model of others [19, 20, 47]. Intriguingly, signals of cooperativeness might even have a paradoxical effect in those with BPD, since they showed less prosocial behavior than HC when interaction partners behaved cooperatively [54], or when oxytocin was administered to facilitate trust [57, 58].

Although our study had a number of strengths, including a large sample size, a well-validated experimental paradigm, and the first examination of this important social-cognitive process in BPD patients, a number of limitations have also to be acknowledged. Although our results point to memory biases in social cognition in BPD, future studies should also address the question of diagnostic specificity, since we did not include a clinical control group. Relatedly, our BPD sample had a lot of comorbid diagnoses, which is common in BPD samples (e.g., [59]), but also entails the possibility that both internalizing and externalizing spectra disorders might moderate the observed effects, or memory biases are caused by a general factor of disorder severity [60]. Consequently, we do not assume that the observed bias is unique for BPD, because personality disorders are generally marked by maladaptive cognitive schemas and interpersonal problems [29]. Transferring our results into the dimensional model in the current version of the International Classification of Diseases (ICD-11), the detachment trait domain is most likely related to negative attitudes towards others, and therefore should be investigated as a mediator for biases in social cognition [61].

## Conclusions

In this study, we experimentally tested memory for cooperative and uncooperative interaction partners in BPD, and detected difficulties to remember cooperative interaction partners. We did not detect any differences between groups with regard to the effects of a-priori trustworthiness. We replicated previous findings that those with BPD showed deficits in cooperation [8], especially when they interacted with trustworthy targets. Both may result in fewer opportunities to cooperate with trustworthy interaction partners, thereby maintaining maladaptive cognitive schemas [19, 20], reduced trust [7], and low interpersonal functioning [1, 2] in BPD.

## Data Availability

The methods of our experiment as well as all datasets supporting the conclusions of this article are available in the OSF repository (https://osf.io/hvf42/).

## Footnotes

1 We re-run all analyses of the memory test entering dimensional scores of trustworthiness from the web-study as a predictor, which did not change the results.
